# Identifying Mitochondrial-Related Genes NDUFA10 and NDUFV2 as Prognostic Markers for Prostate Cancer through Biclustering

**DOI:** 10.1155/2021/5512624

**Published:** 2021-05-22

**Authors:** Haokun Zhang, Yuanhua Shao, Weijun Chen, Xin Chen

**Affiliations:** ^1^Guangdong Key Laboratory of IoT Information Technology, School of Automation, Guangdong University of Technology, Guangzhou 510006, China; ^2^School of Computer Science and Engineering, Sun Yat-sen University, Guangzhou 510006, China

## Abstract

Prostate cancer is currently associated with higher morbidity and mortality in men in the United States and Western Europe, so it is important to identify genes that regulate prostate cancer. The high-dimension gene expression profile impedes the discovery of biclusters which are of great significance to the identification of the basic cellular processes controlled by multiple genes and the identification of large-scale unknown effects hidden in the data. We applied the biclustering method MCbiclust to explore large biclusters in the TCGA cohort through a large number of iterations. Two biclusters were found with the highest silhouette coefficient value. The expression patterns of one bicluster are highly similar to those found by the gene expression profile of the known androgen-regulated genes. Further gene set enrichment revealed that mitochondrial function-related genes were negatively correlated with AR regulation-related genes. Then, we performed differential analysis, AR binding site analysis, and survival analysis on the core genes with high phenotypic contribution. Among the core genes, NDUFA10 showed a low expression value in cancer patients across different expression profiles, while NDUFV2 showed a high expression value in cancer patients. Survival analysis of NDUFA10 and NDUFV2 demonstrated that both genes were unfavorable prognostic markers.

## 1. Introduction

Prostate cancer has a very high morbidity and mortality rate in men in the United States and Western Europe. Approximately 29% of patients who die of prostate cancer are 30-40 years old, and approximately 64% of them are 60-70 years old [1]. Therefore, the identification of genes related to the development and progression of prostate cancer is of great significance for the treatment of prostate cancer. Androgen and androgen receptor (AR) play an important role in the growth of the prostate, the maintenance of efficacy, and the occurrence and progression of prostate cancer [2].

The principle of the biclustering algorithm is to select a subset of rows and columns from a data set and utilize specific measures to maximize the quality of a bicluster, which was first applied to gene expression by Cheng and Church [3]. A gene expression profile typically contains around 20,000 genes and dozens to hundreds of samples. Based on the gene expression profile, the coexpression analysis method was used to detect gene modules related to prostate cancer. Genes that are highly similar to the known androgen regulation genes are regarded as prostate cancer-related genes. Therefore, a clustering method is needed to identify the coexpressed gene modules. At present, it has been found that biclustering has been proven to be a NP-hard problem [4]. A huge number of existing biclustering algorithms involve disparate quality measures and search heuristics to explore gene modules [5]. Mean square residual score is used by various biclustering methods [3], such as MSB [6], FLOC [7], and BiHEA [8]. To serve as a measure of quality, traditional biclustering methods indeed detect biologically related biclusters but only find small rather than large gene coexpression modules [9]. So far, there are many pattern-based biclustering methods, such as PM [10], BicNET [11], and BicPAMS [12]. Most existing algorithms of biclustering perform well in finding various tiny biclusters involving relatively few genes which are highly coexpressed among all the samples, but they have limited ability to acquire a large number of synergistic regulatory genes in a subset of samples.

Bentham et al. developed MCbiclust to identify large biclusters, which provides a novel model-centered unsupervised method to search for a huge number of significant coregulatory genes in a small subset of samples [13]. They have shown that the patterns discovered by this method have biological relevance and significance, which can be used to discover large networks controlled by master transcriptional regulators that may determine basic cellular phenotypes [13].

In this paper, we applied MCbiclust to the RNA sequencing data of prostate cancer in a TCGA cohort. We successfully identified two gene modules by MCbiclust. We then relate the first bicluster to pathological and clinical information which contains biochemical recurrence, new tumor event after initial treatment, number of lymph nodes, and Gleason score. Next, we compared the two biclusters with the biclusters detected using known androgen regulatory genes. Fortunately, we found that the expression patterns of one bicluster were highly similar to those acquired through known ARGs. Moreover, we carried out gene enrichment analysis for the two biclusters. Finally, we identified the core genes with negative correlation in mitochondrial functions by GSEA. Further, differential analysis, survival analysis, and ChIP-seq analysis revealed that both NDUFV2 and NDUFA10 were involved in AR regulation.

## 2. Materials and Methods

### 2.1. Gene Expression Profile

The data used in this paper was RNA-seq data for prostate cancer from a TCGA cohort, with a total of 20,424 genes and 437 samples. Those genes whose expression value was smaller than one in at least one sample were filtered out. Consequently, 6,755 genes were used for further analysis. Then log10 transformation and *Z*-score standardization were performed to obtain the normalized expression profile. The known androgen-regulated genes we used were obtained from ARGDB [14]. We mapped these genes to the gene expression profile from the TCGA cohort, and eventually, we obtained a gene expression profile containing 859 matched genes and 437 samples.

### 2.2. Seed Sample Selection

The first step in using MCbiclust, as shown in Figure S1A, is to look for the subset of the samples that has the highest correlation for the selected genes, which is achieved by calculating the absolute average of the correlation values. Mathematically, for the gene expression profile measuring multiple gene probes across multiple samples, let *X* represent the set of all gene probes and *Y* represent the set of all samples. Then, define the subsets of *X* and *Y*, such as *I* ⊂ *X* and *J* ⊂ *Y*. Subsets *I* and *J* form a bicluster, the strength of which is measured by the correlation between paired probes in set *I*. The correlation between probe *i* ∈ *I* and probe *k* ∈ *I* in sample set *J* is denotable as *C*_*i*,*k*_^*J*^. In addition, |*I*| stands for the number of genes in set *I*. The intensity of biclustering is represented by score *α*, as shown in

(1) $$\begin{array}{c} \alpha_{I}^{J}=\frac{1}{{\left|I\right|}^{2}}\underset{i\in I}{\sum }\underset{k\in I}{\sum }\text{a}\text{bs}\left(C_{i,k}^{J}\right), \end{array}$$

where the abs function represents the absolute value, and the *α* function represents the mean value of the absolute gene-gene correlation calculated from the matrix of gene probe set *I* and the sample set *J*. The high *α*_*I*_^*J*^ value indicates that the gene probe in *I* is becoming a strong regulator in sample set *J*. The value of *α*_*I*_^*J*^ is calculated using the absolute value of *C*_*i*,*k*_^*J*^, and these probes can be positively or negatively correlated. This was achieved by first obtaining a sample seed containing relatively small samples of very high *α*_*I*_^*J*^ values. This step is implemented by initially selecting a random subset of samples and repeating multiple times. During the iterations, a subset of samples with higher *α*_*I*_^*J*^ value will substitute the original samples. According to MCbiclust, good results can be obtained from 1,000 iterations. We used 1,500 iterations to ensure a good result. Finally, the results can be presented by calculating the correlation matrix and drawing the heat map.

### 2.3. Identifying Gene Modules (Bicluster)

Once the seed samples have been selected, as shown in Figure S1B, it is possible to screen all the relevant modules through principal component analysis (PCA). The first principal component (PC1) is calculated as the component that explains the largest variance in the data. Therefore, PC1 will be the variable to summarize this correlation among the discovered biclusters with strong correlation. Then, we performed sample selection based on the last 10% of the sorted samples, which are assumed not to belong to the bicluster. Once such a threshold of PC1 for biclusters is found, it is important to align the PC1 vector and the correlation values (CV) correctly.

The steps described above are based on a single cycle. At the same time, in order to optimize the calculation effect, we performed 1,500 iterations for each cycle to get a better seed. In addition, we performed 1,000 cycles to obtain different good seeds for subsequent classification.

### 2.4. Verifying the Correlation between Biclusters and the Regulation of Prostate Cancer

Through the silhouette coefficient, the biclustering result is the best with two biclusters. Next, we also performed biclustering based on the expression profile of the known androgen regulatory genes, which obtain a bicluster related with androgen regulation.

### 2.5. GO Enrichment Analysis of the Genes in the Biclusters

We performed gene set enrichment for the genes in the biclusters, utilizing the built-in function GOEnrichmentAnalysis in MCbiclust. This function also used the average CV as an input parameter. GO terms were ranked according to the *p* value adjusted by the Bonferroni method.

### 2.6. Assessing the Contribution of Genes to Phenotypes by GSEA

Then, we used GSEA [15, 16] for gene set enrichment analysis to assess the distribution trend of genes from a predefined gene set in a gene list ordered by phenotype relevance. Then, we determine their contribution to the phenotype and define the genes with high phenotypic contribution as core genes. The input data consists of two parts: one is the gene set obtained by biclustering, and the other is the gene sorted according to CV.

### 2.7. Differential Analysis of Candidate Genes

We used the M-W *U* test for differential analysis of candidate genes regulated by AR across multiple expression profiles. We set the threshold of *p* value at 0.05, and extracted the genes with significant differences in multiple expression profiles for further analysis.

### 2.8. Analyzing AR Binding Sites in Candidate Genes by ChIP-Seq

ChIP-seq was used to determine whether the candidate genes contained the AR binding sites. ChIP-seq data (GSE28951 from the GEO database) were aligned to the reference human genome (UCSC, hg19). Binding peaks were determined using Control-Based ChIP-seq Analysis Tools with reference to a set of input reads as negative control [17]. Peaks were defined with a stringent cutoff (FDR ≤ 0.005).

## 3. Results

### 3.1. Two Biclusters Were Identified with the Highest Silhouette Coefficient

MCbiclust was developed to explore biclusters with a large number of genes. However, its computation efficiency decreases when the gene number exceeds 1,000. Therefore, we take the run on a subset of 1,000 genes as an example. The CV of the seeds found by 1,500 iterations is 0.596 and that of the random seeds is 0.362. The difference can be observed between Figure S2A and Figure S2B. In Figure S2B, there are two distinct positive correlation blocks (white) and two distinct negative correlation blocks (red), which are very intuitive. Compared with Figure S2A, the seeds detected by 1,500 iterations in Figure S2B have better performance, and it can be seen that there are two obvious positive correlation blocks along the diagonal. It is clear that the seeds generated from multiple iterations are much better. It is demonstrated in Figure S2C that after using hierarchical clustering to optimize the seeds, the color inside the block is the purest, which proves that by using optimized seeds, we can get a gene cluster with high correlation. Among them, the more obvious blocks the heat maps have, the better are the seeds that are chosen (Figure S2)).

To improve computing efficiency, we divided the expression profiles of 6,755 genes into seven subsets that contained no more than 1,000 genes. We run on each subset in the multiple-run simulation. 2,988 genes were randomly selected by screening the absolute correlation value of more than 60% expression value which was greater than or equal to 0.9.

**Figure 1:**
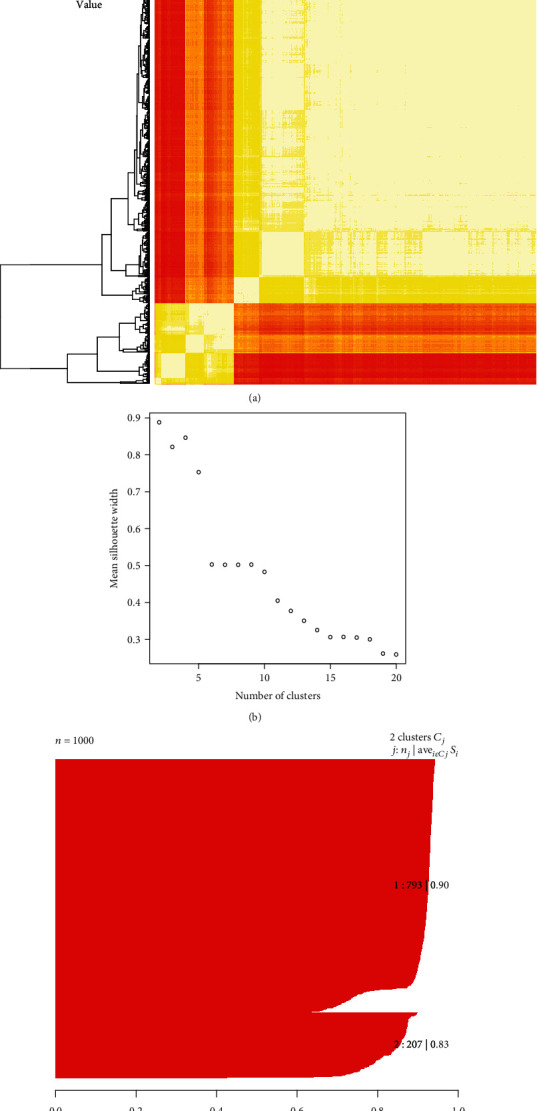
Two biclusters identified by multiple runs with the highest silhouette coefficient. (a) The red part represents low correlation, and the white part represents high correlation. The horizontal and vertical coordinates of the heat map represent the 1st cycle and the 1000th cycle. (b) Mean silhouette width under different numbers of biclusters. (c) The silhouette coefficient of two biclusters. The two red parts below show the distribution of two clusters, and the average silhouette width is 0.89.

After a simulation with multiple runs of up to 1,000 times with 1,500 iterations per time, it was obvious that this heat map had a strong local correlation. In Figure 1(a), the red part represents weakly positive correlation, and the white part represents highly positive correlation. This heat map is different from that in Figure S2 which was based on gene-gene correlation. In Figure 1(a), the rows and columns were changed to the number of cycles. We can see from the heat map (Figure 1(a)) that the results of a multiple-run can be mainly divided into two patterns, and we also observe the small blocks inside the big blocks.

Then, we need to determine the number of biclusters and identify the best number of biclusters. In Figure 1(b), the maximum number of biclusters is set to 20, and as the number of bicluster increases, the silhouette coefficient gradually decreases, which means that when we set the number of biclusters to two, we can obtain the highest score. In Figure 1(c), we can see that the silhouette coefficient value of the two biclusters is 0.90 and 0.83, respectively. The average silhouette coefficient value is 0.89.

### 3.2. Associating the First Bicluster with Pathological and Clinical Information

Two biclusters were detected. Then, we associated the biclusters with pathological and clinical information. In order to infer the biological significance of the results, we analyzed the sampling unit distribution in the bicluster discovered by principal component analysis (PCA). Obviously, although we get two biclusters, the fork graph of the second bicluster is highly deviated (Figure S3), so only the fork graph (Figure S4) of the first bicluster is discussed here. Pathological information including tumor size, number of lymph nodes, Gleason score, tumor activity after initial treatment, and recurrence were analyzed.

**Figure 2:**
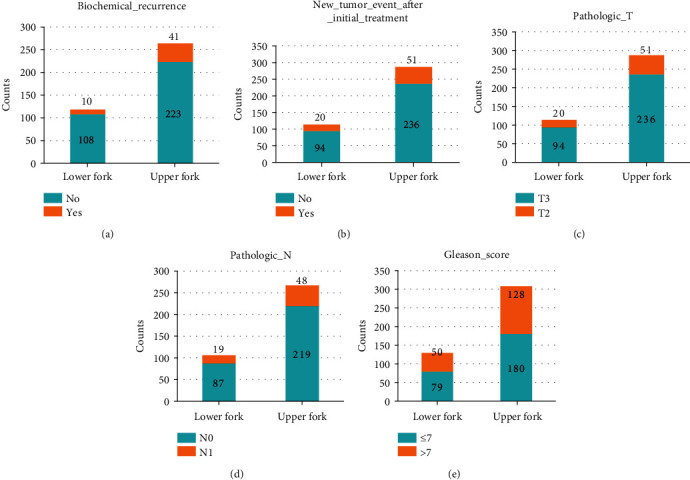
Pathological and clinical information of the first bicluster. The abscissa represents the positive and negative values of PC1 of the sample. Positive PC1 is defined as the upper fork, and negative PC1 is defined as the lower fork. (a) The number of patients with different biochemical recurrence status. Blue represents no recurrence, and orange represents recurrence. (b) The number of patients with different tumor activities after initial treatment. Blue represents inactive, and orange represents active. (c) The number of patients with different levels of tumor sizes. Blue represents the tumor size of T3 level, and orange represents the T2 level. (d) The number of patients with different lymph nodes. Blue represents no lymph nodes, and orange represents the N1 level. (e) The number of patients with a different Gleason score. Blue represents a Gleason score no greater than seven, and orange represents a Gleason score greater than seven.

As shown in Figures S3 and S4, we can obtain a forked pattern based on PC1 and CV. Then, we use a stack bar diagram (Figure 2) to analyze it directly, in which samples are divided into groups with high or low PC1 values. These PC1 values are mainly defined by the average expression level of gene sets.

Figure 2(a) shows that the number of samples without recurrence was larger than that with recurrence, both on the upper and the lower forks. In the upper fork, samples with no recurrence accounted for 84.5% (223/264), and in the lower fork, the proportion was 91.5% (108/118). In Figure 2(b), in the samples after initial treatment, the number of samples with no tumor activity was higher than that with tumor activity. In the upper fork, the proportion of nontumor active samples was 82.2% (236/287), and in the lower fork, the proportion was 82.5% (94/114). As shown in Figure 2(c), in general, the number of samples with T3 grade tumors was larger than that with T2 grade tumors. In the upper fork, the proportion of T3 grade tumor samples was 59.5% (175/294), and in the lower fork, the proportion of T3 grade tumor samples was 59.5% (75/126). In Figure 2(d), the proportion of lymphocyte N0 in all samples was the largest. In the upper fork, the number of N0 samples accounted for 82.0% (219/267), and in the lower fork, the number of N0 samples accounted for 82.1% (87/106). We also associated genes in the first bicluster with the Gleason score. As can be seen from Figure 2(e), samples with a Gleason score ≤ 7 account for the majority. 58.4% (180/308) of the samples in the upper fork have aGleason score ≤ 7, while 61.2% (79/129) of the samples in the lower fork have aGleason score ≤ 7.

### 3.3. The Biclusters Are Related with Androgen Regulation

To further validate the biclusters, known AR-regulated genes (ARGs) of prostate cancer were introduced. Gene expression profiles of known ARGs were comprised of 859 genes and 437 samples. Similar to the processing of 2,988 genes, we performed 10,000 iterations for the known ARGs. Then, the two biclusters previously obtained from multiple iterations were compared with the bicluster obtained from the ARGs.

**Figure 3:**
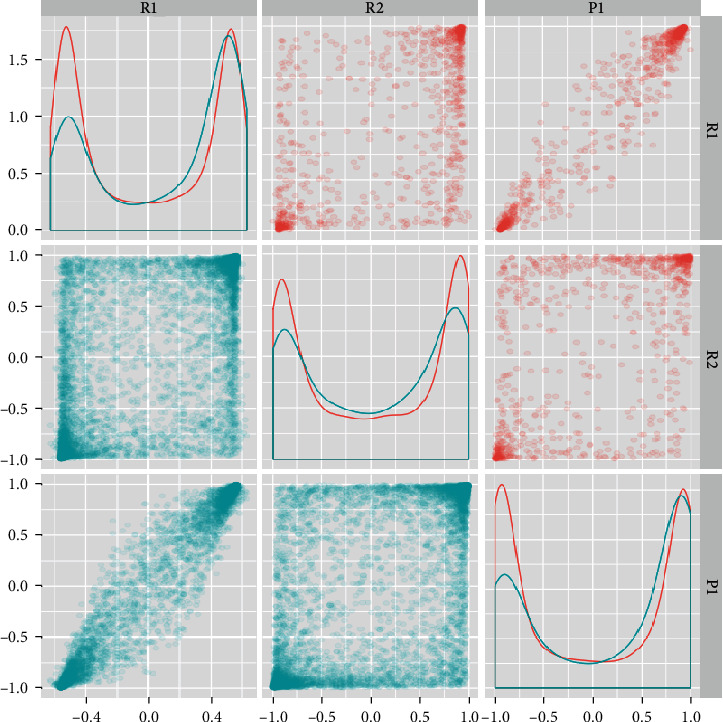
Comparing two biclusters with the bicluster found from known AR genes. In the scatter plot, the horizontal and vertical coordinates are correlation coefficients. Blue represents ARGs, and red represents non-ARGs. R1 and R2 are two biclusters found by a random seed, and P1 is the bicluster identified by known ARGs.

As shown in Figure 3, the horizontal and vertical coordinates are correlation coefficients, where R1 and R2 are two biclusters obtained from multiple random runs, and P1 is the bicluster calculated from the known ARGs. It can be seen from Figure 3 that R1 is highly similar to P1, which proves that we have successfully acquired the set of highly correlated prostate cancer-related genes through randomly selecting sample seed, and it is highly similar with the known regulatory genes.

### 3.4. Mitochondrial-Related Genes Negatively Correlate with AR Regulation

To further analyze the function of genes in the bicluster R1, we used a built-in function in MCbiclust and set adjusted *p* value filtering (*p* ≤ 0.05) to carry out gene enrichment.

**Figure 4:**
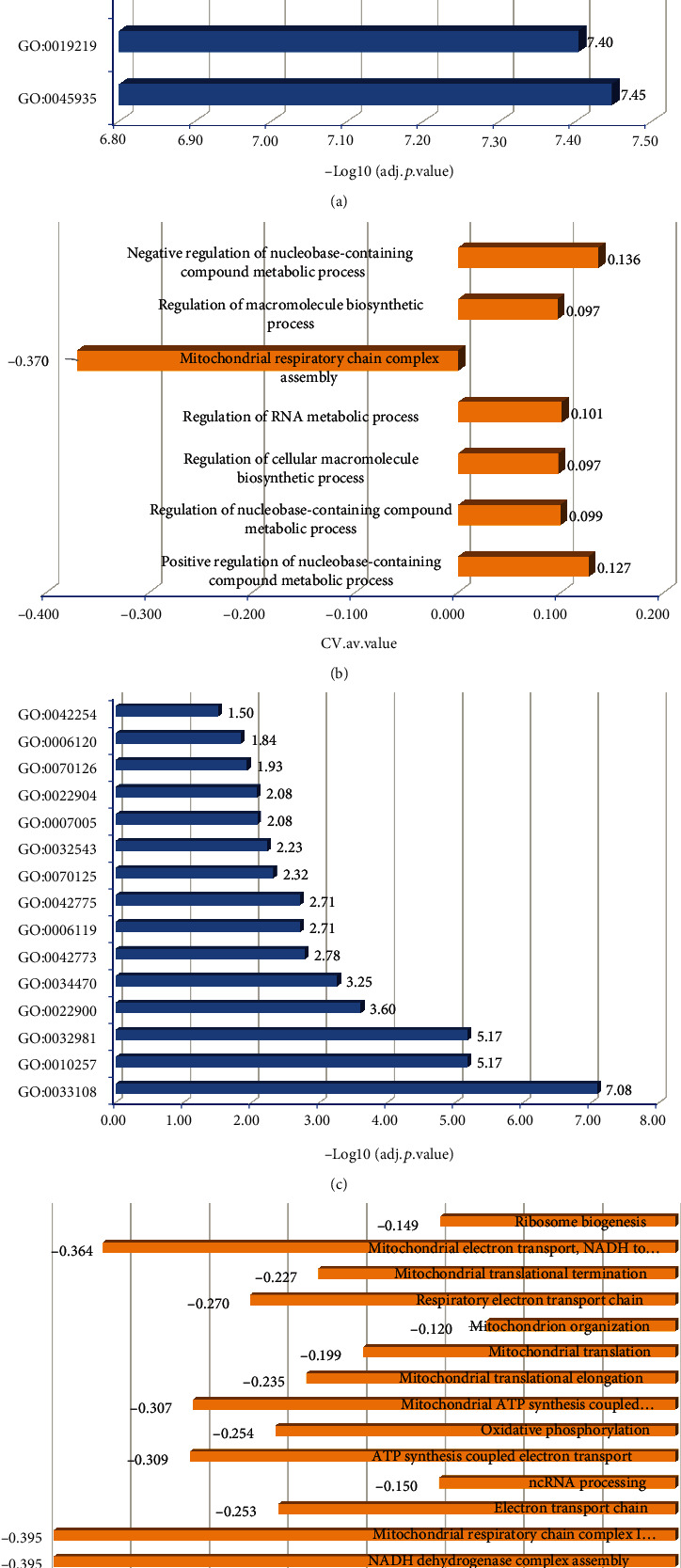
Gene enrichment of the first bicluster. (a) The adj.*p*.values of seven of the most significant GO terms. (b) The average CV for genes in each of the seven terms. (c) The adjusted *p* value of the negative regulation terms. (d) The average CV for genes in the negative regulation terms.

Through the analyses of Figures 2 and 3, we can see that the first bicluster has a better performance; therefore, we mainly discuss the gene enrichment results of the first bicluster. For the genes in the first bicluster R1, there are 265 significant functions (adjusted *p* value ≤ 0.05). We selected the top seven BP terms with an adjusted *p* value smaller than 1.0*e*‐09 for discussion (Figure 4(a)). The seven functions (Figure 4(b)) are regulating a metabolic process with nuclease (positive correlation), regulating metabolic processes with nuclease (positive correlation), control of the macromolecular biosynthesis cells (positive correlation), RNA metabolism regulation (positive correlation), mitochondrial respiratory chain complex assembly (negative correlation), macromolecular biosynthesis regulation (positive correlation), and negative control of the metabolic process with nuclease (positive correlation), respectively.

In the seven functions (Figure 4(b)), we discovered that only the mitochondrial function has a negative correlation. We found that only 15 of the 172 BP functions are negative correlation functions (Figure 4(c)), and among them, 53.3% (8/15) of the functions were related with mitochondrial regulation (Figure 4(d)).

### 3.5. GO Terms with High Negative Enrichment Signal Strength Were Validated by GSEA

**Figure 5:**
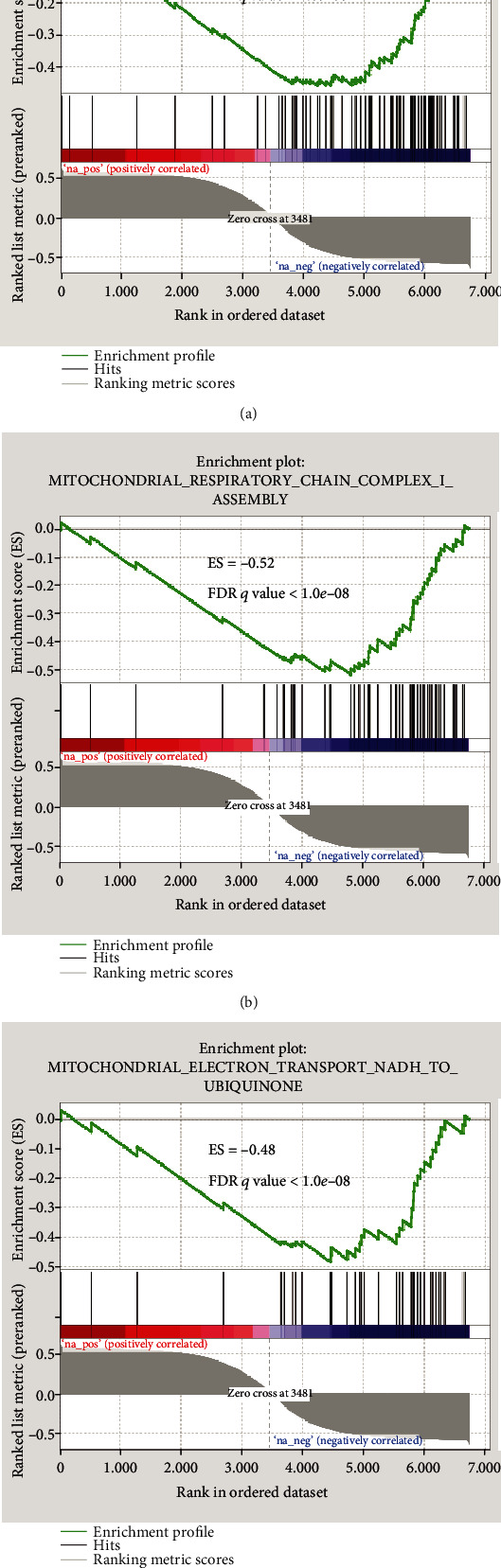
Gene set enrichment analysis of the three terms with negative CV. We can see the enrichment scores of the three terms with negative CV. The genes are ranked according to their CV in descending order.

Since we noticed that mitochondrial functions have a negative CV, we then focused on all the GO terms which have a negative CV (Figures 4(c) and 4(d)). We selected all of the eight GO terms related to mitochondrial function from the negatively correlated GO terms, and extracted the matched genes from each GO term to form a predefined database of gene sets. As shown in Figure 5 and Figure S5, all the eight gene sets exhibited significant performance, among which the ES values of Figures 5(a)–5(c) are less than or equal to -0.46 and the FDR*q*values of terms are both less than1.0*e*‐08. In the following, we use differential analysis and ChIP-seq to further analyze the core genes in these three gene sets.

### 3.6. Six Candidate Genes Identified by Differential Analysis and ChIP-Seq Data

Core genes in the three terms which were mentioned in Figure 5 were screened to form a new gene expression profile, and 29 differential genes with significant expression changes between normal and tumor samples were screened by *U* test in the TCGA cohort (*p* ≤ 0.05). We then focused on the transcription of differentially expressed genes in the process of AR regulation by using ChIP-seq, and finally screened out six genes that have AR binding sites (SAMM50, NDUFA10, SDHAF4, OXA1L, NDUFS5, and NDUFV2). We then introduced six new prostate cancer-related datasets from the GEO database and screened the candidate genes for differential analysis in each dataset.

**Figure 6:**
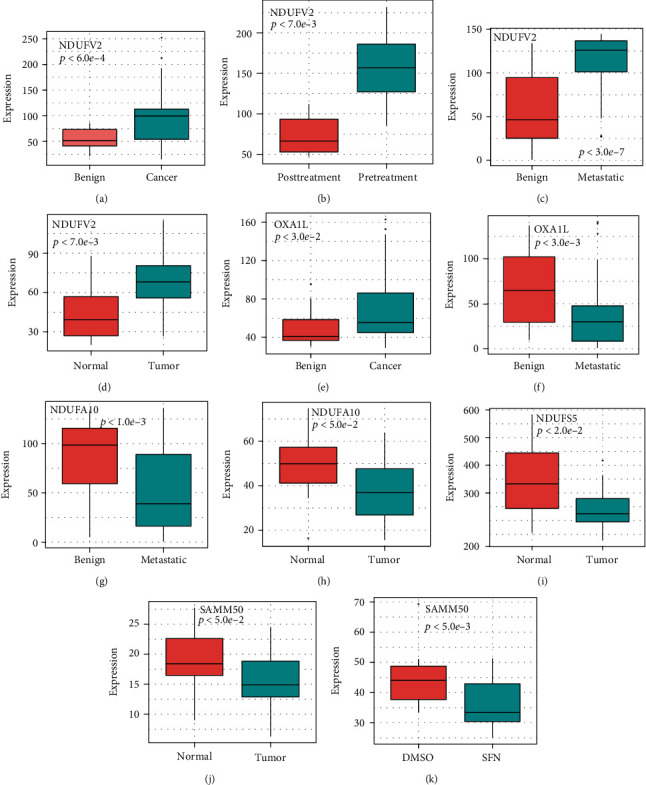
Differential analysis for candidate genes across multiple cohorts. The expression of NDUFV2 in dataset (a) GSE25183 (benign vs. cancer), (b) SRP002628 (posttreatment vs. pretreament), (c) SRP011439 (benign vs. metastatic), and (d) SRP026387 (normal vs. tumor). The expression of OXA1L in (e) GSE25183 (benign vs. cancer) and (f) SRP011439 (benign vs. metastatic). The expression of NDUFA10 in (g) ERP000550 (benign vs. metastatic) and (h) SRP011439 (normal vs. tumor). (i) The expression of NDUFS5 in ERP000550 (normal vs. tumor). The expression of SAMM50 in (j) ERP000550 (normal vs. tumor) and (k) GSE48812 (DMSO treated vs. SFN treated).

NDUFV2 had significant expression differences in the four cohorts. OXA1L, NDUFA10, and SAMM50 were significantly different in the two cohorts. The expression values of NDUFV2 in normal samples were lower than that in tumor samples (Figures 6(a)–6(d)). OXA1L showed inconsistent trends in the tumor and metastatic samples (Figures 6(e) and 6(f)). The expression values of NDUFA10 in normal samples were higher than that in tumor samples (Figures 6(g) and 6(h)). NDUFS5 was significantly different in ERP000550 (Figure 6(i)). SAMM50 (Figures 6(j) and 6(k)) showed higher expression values in normal samples than in cancer samples.

In the above analysis, we conclude that the expression values of NDUFV2 were higher in prostate cancer and NDUFA10 and SAMM50 expression values were lower in tumor samples than in normal samples. The discovery may lead to a new direction in targeted therapy for prostate cancer.

### 3.7. NDUFA10 and NDUFV2 Are Candidate Prognostic Markers

**Figure 7:**
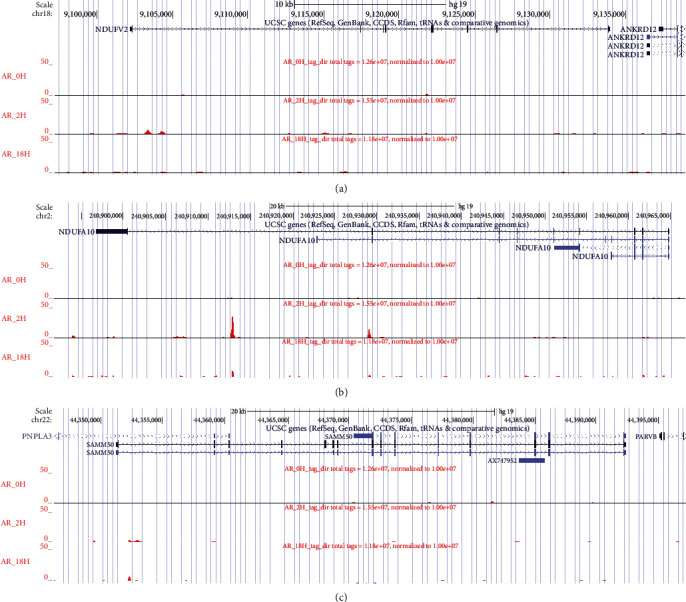
The AR binding site of candidate genes detected by ChIP-seq. The androgen response of candidate genes (a) NDUFV2, (b) NDUFA10, and (c) SAMM50 during the androgen stimulus (zero hour, two hours, and 18 hours).

The binding sites of NDUFV2, NDUFA10, and SAMM50 during AR regulation can be obtained by ChIP-seq. We can see that NDUFV2 (Figure 7(a)) and NDUFA10 (Figure 7(b)) bind to AR at two hours. SAMM50 weakly binds to AR regulation at two hours and 18 hours (Figure 7(c)).

**Figure 8:**
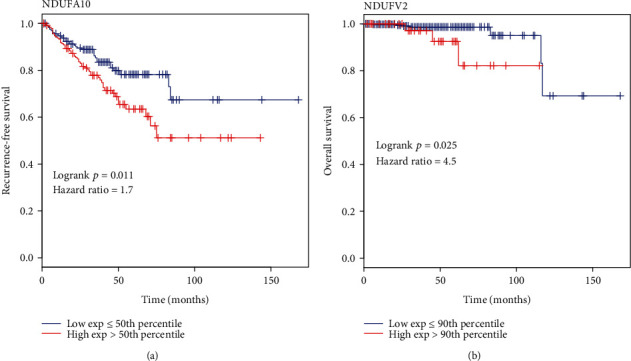
Survival analysis of NDUFA10 and NDUFV2. The abscissa is the time (month), and the ordinate is the RFS or OS. (a) The RFS of NDUFA10 in the TCGA cohort. (b) The OS of NDUFV2 in the TCGA cohort. Blue represents the patient group with a low expression value, and red represents the group with a high expression value.

Next, NDUFA10 and NDUFV2 were analyzed for their prognostic potential using GEPIA2. We found that patients with the high NDUFA10 expression group had a significantly lower recurrence-free survival (RFS) rate (*p* = 0.011) than (Figure 8(a)) the low NDUFA10 expression group. In addition, the high NDUFV2 expression group has shorter overall survival (OS) (*p* = 0.025) than the low NDUFV2 expression group (Figure 8(b)).

## 4. Discussion

MCbiclust is a novel biclustering method for a large-scale expression matrix which is superior for biclustering large numbers of genes. In general, MCbiclust is an algorithm involving iterations, and the more times it is run and iterated, the better will be the obtained result. WGCNA [18] can be applied to scenes with data of high dimensions. However, WGCNA searches for global coexpression of genes across all samples, while MCbiclust [13] is aimed at screening the coexpression of genes in subsets of samples. The goals of dimension reduction methods, such as PCA, ICA [19], or t-SNE [20], are fundamentally different from that of MCbiclust. Although MCbiclust is not particularly friendly to high-dimensional genes, it can also perform a good biclustering of sample subsets through a random grouping process and threshold screening. It can pick out the sets of genes that are highly correlated with prostate cancer from a random seed selection which can reduce the error caused by manually selected parameters.

The MCbiclust algorithm mainly consumes time to explore the theoretically best sample seed, and there are no other parameters that can be invoked for parallel computation in this process. To improve the efficiency of seed searching, we divided the gene expression profile into seven subsets. The gene expression profiles were obtained by extracting subsets of locally highly expressed genes from seven random subsets. Then, a seed sample with good performance was obtained by running the algorithm 1,000 times with 1,500 iterations per time for the extracted gene expression profile. Next, we calculated the value of the contour coefficient under different numbers of biclusters. The biclustering effect is at its best with two biclusters. We subsequently associated the first bicluster with clinicopathological information which contains a biochemical recurrence, a new tumor event after initial treatment, a number of lymph nodes, and a Gleason score. We found that, in a biochemical recurrence, the proportion was higher in the upper fork than in the lower fork and the proportion of samples with a Gleason score of less than seven in the upper fork was lower than that in the lower fork. Then, we introduced the known ARGs and detected the expression pattern of one of the biclusters (R1) that we acquired, which was highly similar to that of the bicluster we obtained through the known ARGs. Therefore, we believe that the genes contained in R1 are also genes related to the function of ARGs, which can be candidates for future study. Through gene enrichment, we found that most of the GO terms with negative CV were related to mitochondrial function. After that, we investigated core genes of GO terms with a significant negative enrichment signal through GSEA. Six genes were differentially expressed between normal and prostate cancer tissues across at least two cohorts and simultaneously had AR binding sites through analysis of public ChIP-seq data. Survival analysis demonstrated that NDUFA10 and NDUFV2 are potential prognostic markers with significantly different RFS or OS among patients with high and low expression values. It should be noted that the data we used for bicluster identification was from the public datasets of TCGA. However, no biological cohort was used to validate the biclusters, which may cause some limitations on the results.

Studies have shown that the absence of NDUFV2 leads to the destruction of multipolar-bipolar transition and polarization of neurons in vivo and in vitro [21]. NDUFA10 is a new candidate gene for screening pathogenic mutations in patients with complex I defects [22]. In addition, although SAMM50 did not show significant RFS or OS in survival analysis, other studies have shown that SAMM50 is associated with nonalcoholic fatty liver disease and affects drP1-dependent mitochondrial morphology [23, 24]. The important effect of oxidative phosphorylation (OXPHOS) on the progression of prostate cancer has been reported. An activated OXPHOS has been observed in docetaxel-resistant prostate cancer cells, and inhibition of OXPHOS reduces nicotinamide adenine dinucleotide phosphate (NADPH) coenzyme production in the oxidative phase of the pentose phosphate pathway, suggesting the effectivity of a combination of chemotherapy and OXPHOS inhibition for the therapy of prostate cancer [25]. Besides, compared with benign prostate and localized prostate cancer tissues, the upregulated genes in castration-resistant prostate cancer are significantly enriched in biological functions including OXPHOS which may relate to the production of NADPH coenzymes [26].

In summary, we applied MCbiclust to identify large gene modules in a subset of samples. We successfully identified the gene set associated with the function of ARGs and discovered a difference in the proportion of biochemical recurrence and the Gleason score among the upper fork and the lower fork. Mitochondrial-related genes including NDUFA10 and NDUFV2 participate in androgen regulation and may be candidate prognostic markers, which could be therapeutic targets of prostate cancer.

## Data Availability

The expression profile data used to support the findings of this study have been deposited in the TCGA, SRA, and GEO repositories.

## Supplementary Materials

Supplementary 1 Figure S1: the process of biclustering analysis using MCbiclust. Here, we can clearly see the steps required when using the MCbiclust. Boxes of the same color represent the steps of the same type of function or the results of the same process. (A) The workflow of seed sample selection. (B) The steps of identifying gene modules and further analysis.

Supplementary 2 Figure S2: heat maps drawn with different correlation values which are calculated to get better seeds. The abscissa and ordinate are genes, and the color of each grid represents the correlation value. White represents a strong correlation, and red represents a low correlation. Heat map of the correlation value calculated by (A) a random seed, (B) multiple iterations, and (C) hierarchical clustering of multiple iterations.

Supplementary 3 Figure S3: the fork graph of the second bicluster. The vertical coordinate in the graph represents the value of the principal component, and the horizontal coordinate represents the ranking of the samples. (A) The distribution of patients with different recurrence status. The red dots represent no recurrence, and the blue dots represent recurrence. (B) The distribution of patients with different tumor activities after initial treatment. Red dots represent inactive, and blue dots represent active. (C) The distribution of patients with different tumor sizes of lymph nodes. The red dots represent the tumor size of T2 level, and the blue dots represent the tumor size of T3 level. (D) The distribution of patients with different tumor numbers of lymph nodes. The red dots represent N0 lymph nodes, and the blue represents N1 lymph nodes. (E) The distribution of patients with a different Gleason score. Red dots represent a Gleason score that is less than or equal to 7, and the cyan blue dots represent a Gleason score that is greater than 7.

Supplementary 4 Figure S4: the fork graph of the first bicluster. The vertical coordinate in the graph represents the value of the principal component, and the horizontal coordinate represents the ranking of the samples. (A) The distribution of patients with different recurrence status. The red dots represent no recurrence, and the blue dots represent recurrence. (B) The distribution of patients with different tumor activities after initial treatment. Red dots represent inactive, and blue dots represent active. (C) The distribution of patients with different tumor sizes of lymph nodes. The red dots represent the tumor size of T2 level, and the blue dots represent the tumor size of T3 level. (D) The distribution of patients with different tumor numbers of lymph nodes. The red dots represent N0 lymph nodes, and the blue represents N1 lymph nodes. (E) The distribution of patients with a different Gleason score. Red dots represent a Gleason score that is less than or equal to 7, and the cyan blue dots represent a Gleason score that is greater than 7.

Supplementary 5 Figure S5: gene set enrichment analysis of the five terms with negative CV. The genes are ranked according to their CV in descending order. The enrichment scores of the five terms with negative CV are shown for (A) mitochondrial ATP synthesis coupled electron transport, (B) mitochondrial translation, (C) mitochondrial translational elongation, (D) mitochondrial translational termination, and (E) mitochondrion organization.
