# Supplementary figures and images for: Identifying Mitochondrial-Related Genes NDUFA10 and NDUFV2 as Prognostic Markers for Prostate Cancer through Biclustering
Source: Biomed Res Int. 2021 May 22;2021:5512624. doi: 10.1155/2021/5512624 (PMC8168472; doi:10.1155/2021/5512624)

A

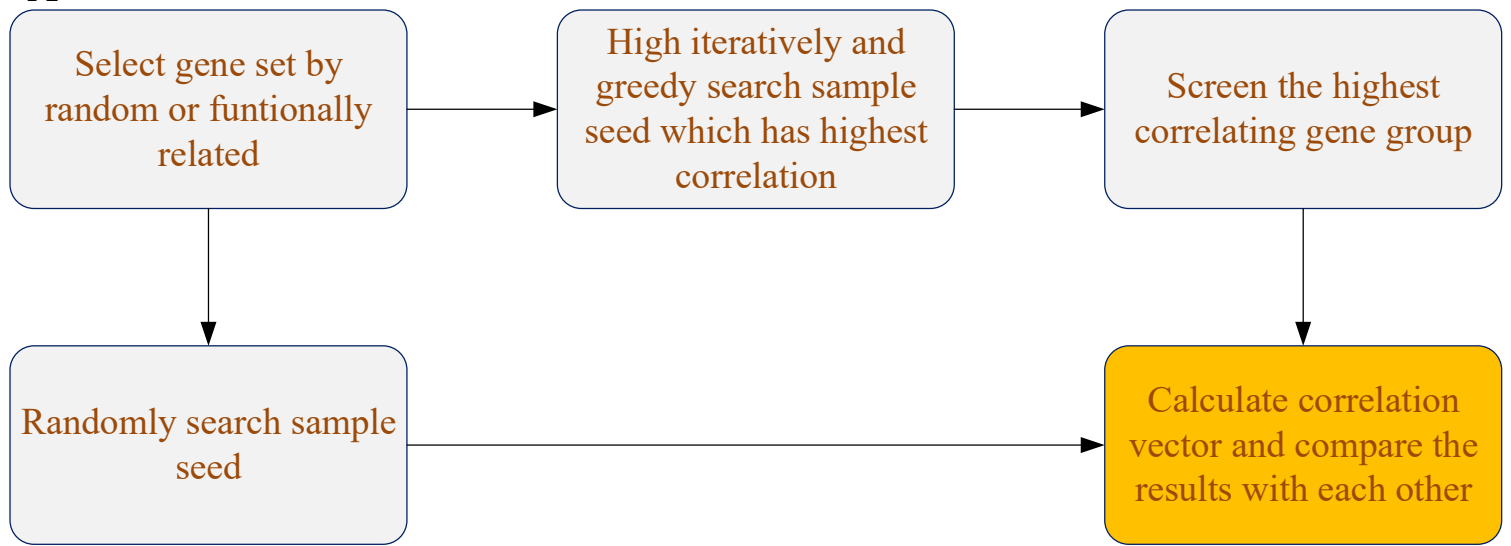

B

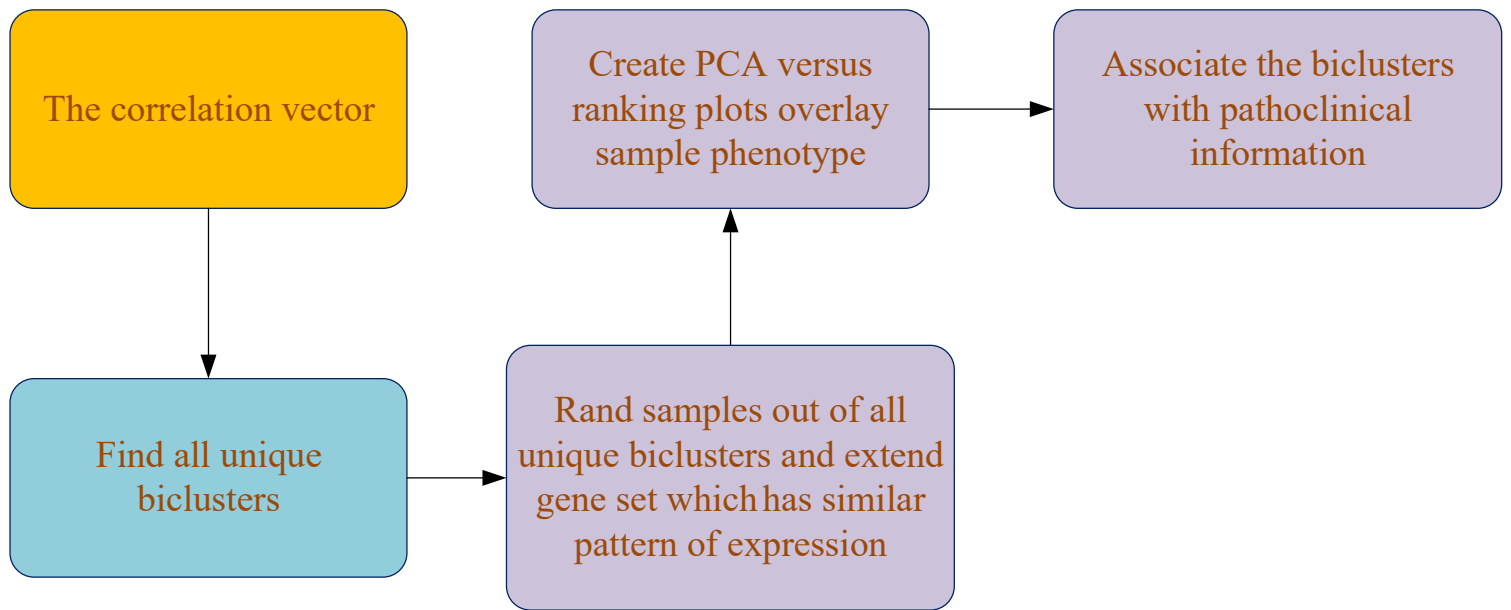

Supplement: Supplementary 1 — Figure S1: the process of biclustering analysis using MCbiclust. Here, we can clearly see the steps required when using the MCbiclust. Boxes of the same color represent the steps of the same type of function or the results of the same process. (A) The workflow of seed sample selection. (B) The steps of identifying gene modules and further analysis. [file 5512624.f1.pdf]

**A**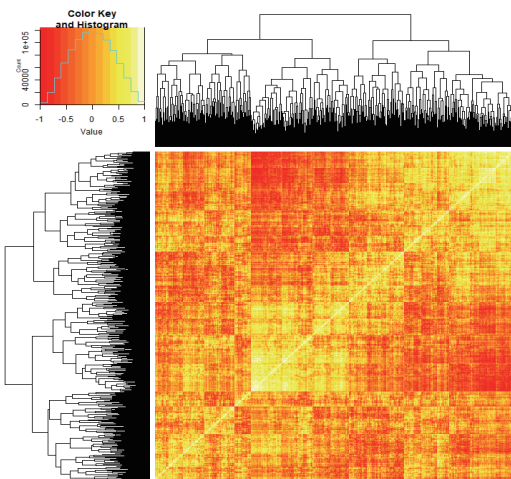**B**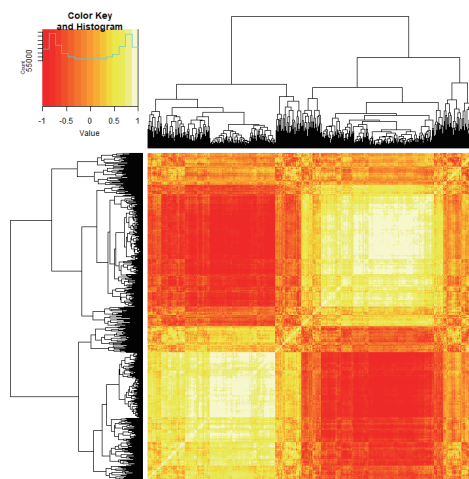**C**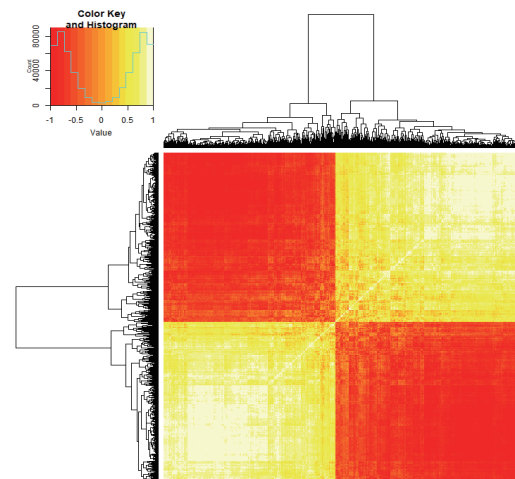

Supplement: Supplementary 2 — Figure S2: heat maps drawn with different correlation values which are calculated to get better seeds. The abscissa and ordinate are genes, and the color of each grid represents the correlation value. White represents a strong correlation, and red represents a low correlation. Heat map of the correlation value calculated by (A) a random seed, (B) multiple iterations, and (C) hierarchical clustering of multiple iterations. [file 5512624.f2.pdf]

**A**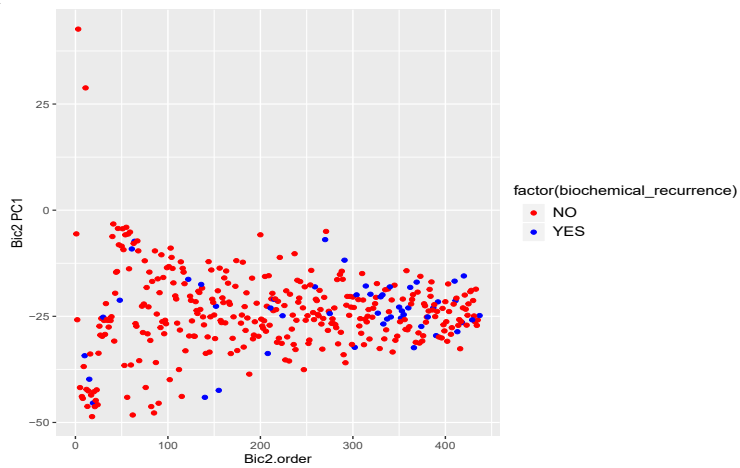**B**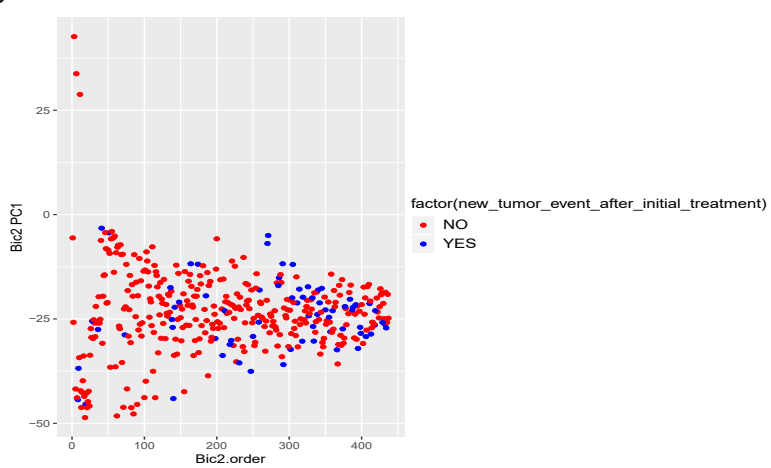**C**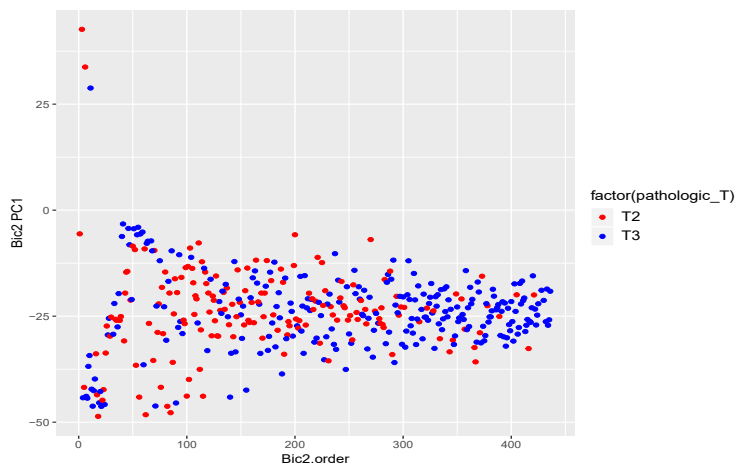**D**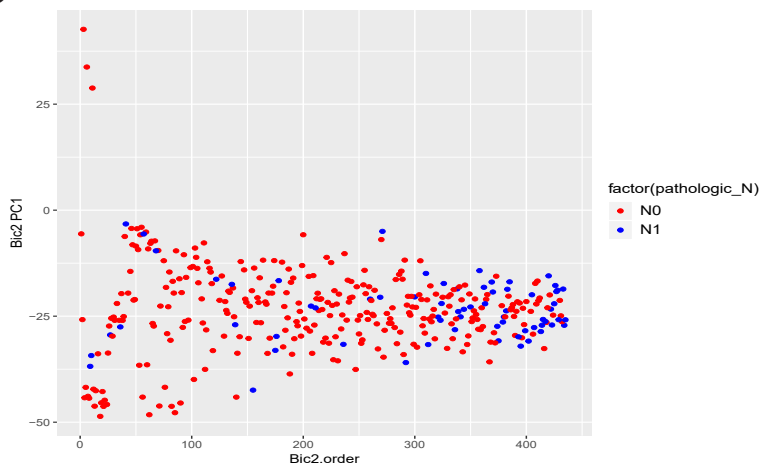**E**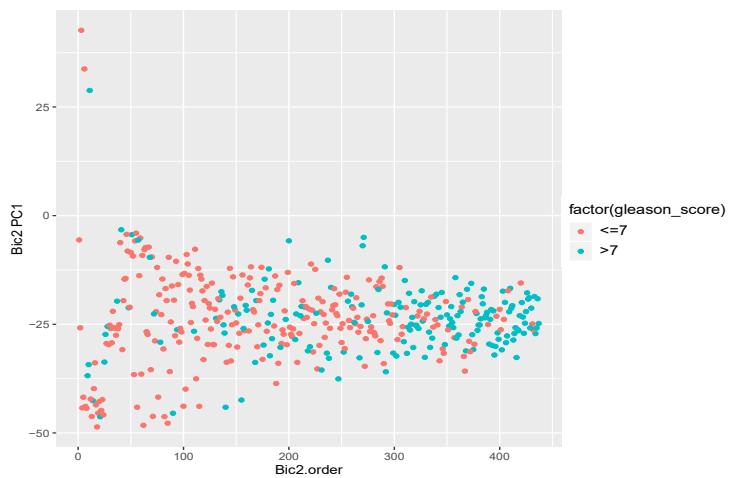

Supplement: Supplementary 3 — Figure S3: the fork graph of the second bicluster. The vertical coordinate in the graph represents the value of the principal component, and the horizontal coordinate represents the ranking of the samples. (A) The distribution of patients with different recurrence status. The red dots represent no recurrence, and the blue dots represent recurrence. (B) The distribution of patients with different tumor activities after initial treatment. Red dots represent inactive, and blue dots represent active. (C) The distribution of patients with different tumor sizes of lymph nodes. The red dots represent the tumor size of T2 level, and the blue dots represent the tumor size of T3 level. (D) The distribution of patients with different tumor numbers of lymph nodes. The red dots represent N0 lymph nodes, and the blue represents N1 lymph nodes. (E) The distribution of patients with a different Gleason score. Red dots represent a Gleason score that is less than or equal to 7, and the cyan blue dots represent a Gleason score that is greater than 7. [file 5512624.f3.pdf]

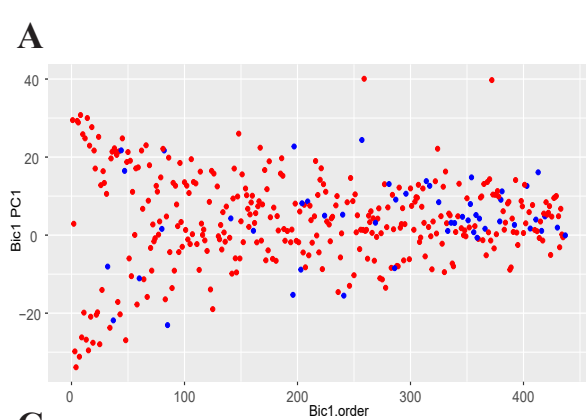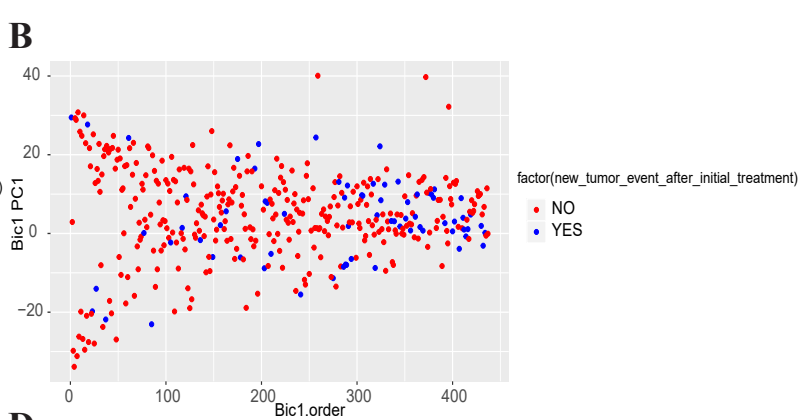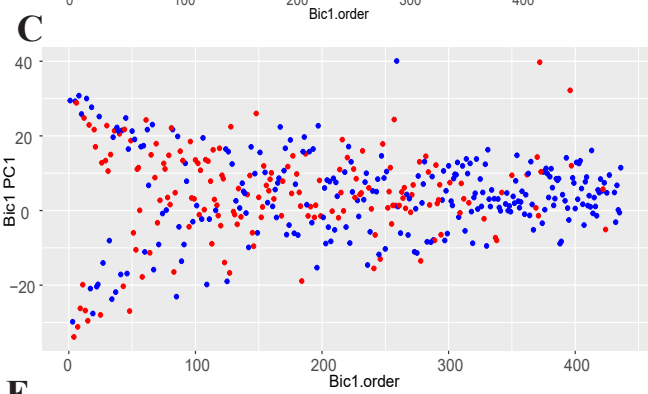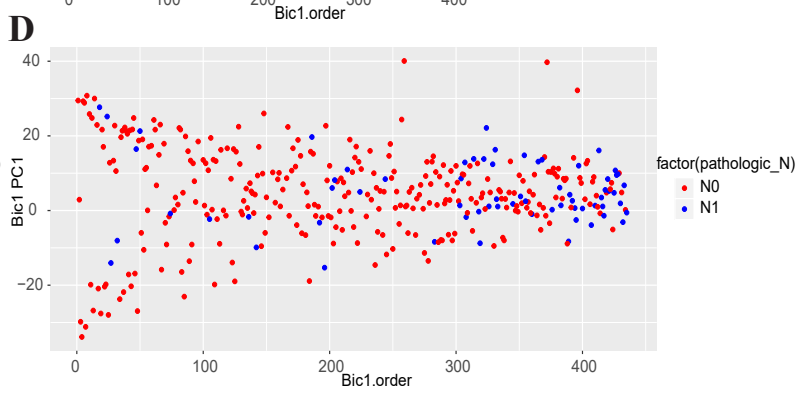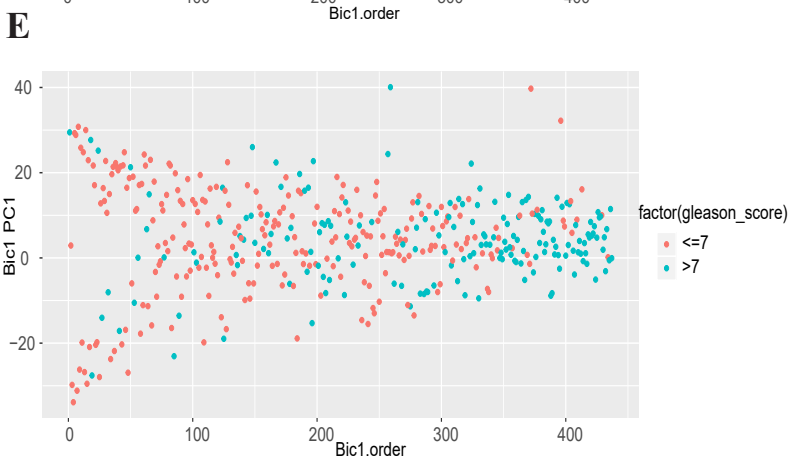

Supplement: Supplementary 4 — Figure S4: the fork graph of the first bicluster. The vertical coordinate in the graph represents the value of the principal component, and the horizontal coordinate represents the ranking of the samples. (A) The distribution of patients with different recurrence status. The red dots represent no recurrence, and the blue dots represent recurrence. (B) The distribution of patients with different tumor activities after initial treatment. Red dots represent inactive, and blue dots represent active. (C) The distribution of patients with different tumor sizes of lymph nodes. The red dots represent the tumor size of T2 level, and the blue dots represent the tumor size of T3 level. (D) The distribution of patients with different tumor numbers of lymph nodes. The red dots represent N0 lymph nodes, and the blue represents N1 lymph nodes. (E) The distribution of patients with a different Gleason score. Red dots represent a Gleason score that is less than or equal to 7, and the cyan blue dots represent a Gleason score that is greater than 7. [file 5512624.f4.pdf]

**A**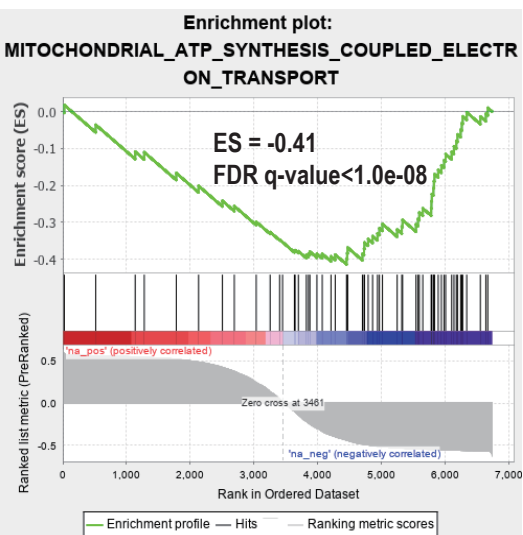**B**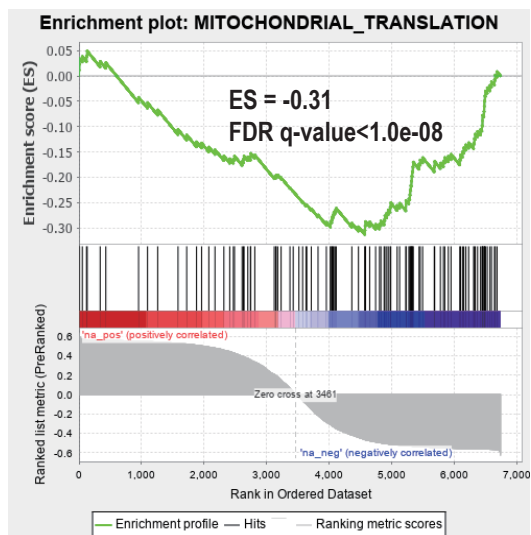**C**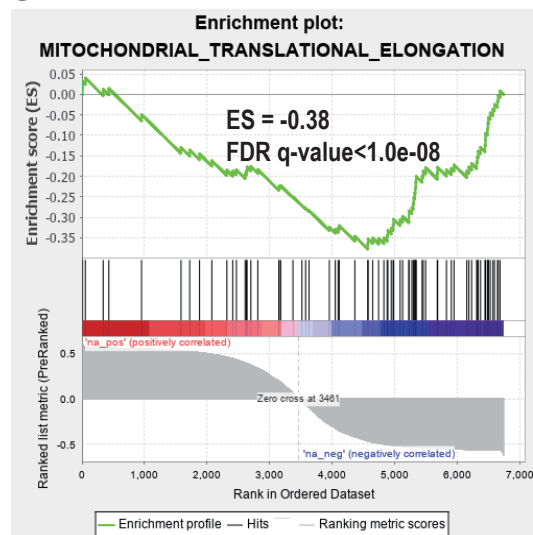**D**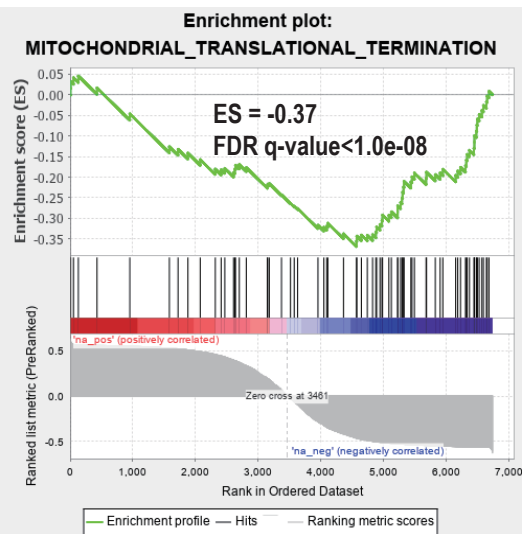**E**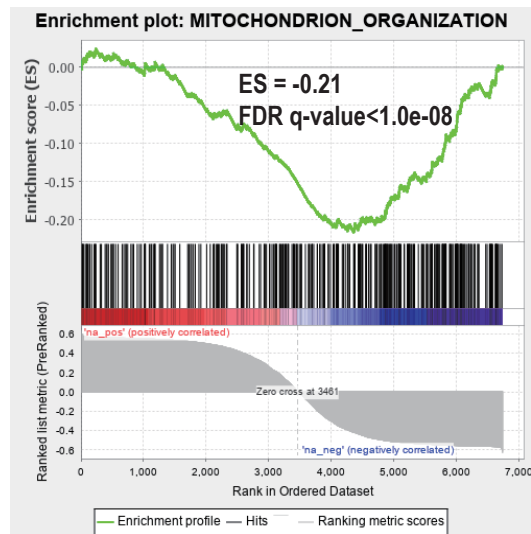

Supplement: Supplementary 5 — Figure S5: gene set enrichment analysis of the five terms with negative CV. The genes are ranked according to their CV in descending order. The enrichment scores of the five terms with negative CV are shown for (A) mitochondrial ATP synthesis coupled electron transport, (B) mitochondrial translation, (C) mitochondrial translational elongation, (D) mitochondrial translational termination, and (E) mitochondrion organization. [file 5512624.f5.pdf]
